# Risk of SARS-CoV-2 Infection Among People Living With HIV in Wuhan, China

**DOI:** 10.3389/fpubh.2022.833783

**Published:** 2022-04-29

**Authors:** Mengmeng Wu, Fangzhao Ming, Songjie Wu, Yanbin Liu, Xiaoxia Zhang, Wei Guo, Gifty Marley, Weiming Tang, Ke Liang

**Affiliations:** ^1^Department of Infectious Diseases, Zhongnan Hospital of Wuhan University, Wuhan, China; ^2^Wuhan Research Center for Infectious Diseases and Cancer, Chinese Academy of Medical Sciences, Wuhan, China; ^3^Wuchang District Center for Disease Control and Prevention, Wuhan, China; ^4^Department of Nosocomial Infection Management, Zhongnan Hospital of Wuhan University, Wuhan, China; ^5^Department of Pathology, Zhongnan Hospital of Wuhan University, Wuhan, China; ^6^Department of Pathology, School of Basic Medical Sciences, Wuhan University, Wuhan, China; ^7^School of Public Health of Nanjing Medical University, Nanjing, China; ^8^Guangdong Second Provincial General Hospital, Guangzhou, China; ^9^University of North Carolina at Chapel Hill Project-China, Guangzhou, China; ^10^Hubei Engineering Center for Infectious Disease Prevention, Control and Treatment, Wuhan, China

**Keywords:** SARS-CoV-2, people living with human immunodeficiency virus (PLWH), IgG, IgM, asymptomatic infectors, symptomatic patient

## Abstract

**Background:**

In the era of the COVID-19 pandemic, people living with HIV (PLWH) face more challenges. However, it is unclear if PLWH is more susceptible to the severe acute respiratory syndrome coronavirus 2 (SARS-CoV-2) infection than HIV-negative individuals. This study aimed to explore the prevalence of the SARS-CoV-2 infection and the associated risk factors among PLWH.

**Methods:**

From 1 to 30 May 2020, we conducted a cross-sectional survey that enrolled 857 PLWH and 1,048 HIV-negative individuals from the Wuchang district in Wuhan, China. Our data analysis compared the rate of the SARS-CoV-2 infection among PLWH and HIV-negative participants, and the proportions of symptomatic patients and asymptomatic infectors between the two groups. We also assessed the risk factors associated with the SARS-CoV-2 infection among PLWH.

**Results:**

Overall, 14/857 (1.6%) PLWH and 68/1,048 (6.5%) HIV-negative participants were infected with SARS-CoV-2. Among the SARS-CoV-2-infected PLWH participants, 6/14 (42.8%) were symptomatic patients, 4/14 (28.6%) were SARS-CoV-2 nucleic acid-positive asymptomatic infectors, and 4/14 (28.6%) were serology-positive asymptomatic infectors. Among the infected HIV-negative participants, 5/68 (7.4%) patients were symptomatic and 63/68 (92.6%) were serology-positive asymptomatic infectors. The rate of the SARS-CoV-2 infection was lower among the PLWH than in the HIV-negative group (1.96% vs. 5.74%, *p* = 0.001) and the rate of morbidity among the symptomatic patients was similar between the two groups (*p* = 0.107). However, there were more serology-positive asymptomatic infectors among the infected HIV-negative participants than among the infected PLWH (0.54% vs. 5.46%, *p* = 0.001). Furthermore, being 50 years or older (aOR = 4.50, 95% CI: 1.34–15.13, *p* = 0.015) and having opportunistic infections (aOR = 9.59, 95% CI: 1.54–59.92, *p* = 0.016) were associated with an increased risk of SARS-CoV-2 infection among PLWH.

**Conclusions:**

PLWH has more varied forms of the SARS-CoV-2 infection than the HIV-negative population and should, therefore, undertake routine screening to avoid late diagnosis. Also, older age (≥50 years) and having opportunistic infections increase the risks of SARS-CoV-2 infection among PLWH.

## Introduction

By 10 December 2021, a total of 268,501,588 confirmed severe acute respiratory syndrome coronavirus 2 (SARS-CoV-2) cases and 5,286,843 SARS-CoV-2-related deaths had been reported globally since the COVID-19 pandemic onset in 2019 (1). As living with HIV compromises natural immunity and could translate to more complications in COVID-19-infected patients, persons living with HIV (PLWH) were considered more vulnerable to the SARS-CoV-2 (2). However, research findings from some recent studies showed that the SARS-CoV-2 infection does not increase morbidity in PLWH (3). In addition, asymptomatic infectors are largely overlooked in the existing literature as most previous studies did not consider them (4, 5). Whether knowledge and evidence on the SARS-CoV-2 infection will remain similar or differ from the existing literature after accounting for these two groups of SARS-CoV-2 infections is currently unknown.

This study aimed to investigate further the prevalence of the SARS-CoV-2 infection and determine its associated risk factors among PLWH in Wuhan, China.

## Materials and Methods

### Study Design and Participants' Recruitment

As an extension of our previous work (6, 7), we conducted a cross-sectional study among two cohorts of people that participated in the previous SARS-CoV-2 seroepidemiological survey in the Wuchang district of Wuhan. From 1 May 2020 to 30 May 2020, we recruited PLWH and HIV-negative individuals aged 18 years and above who had lived in the Wuchang district for at least 1 month during the COVID-19 onset (from 1 December 2019 to 8 April 2020).

### Participant's Recruitment

All PLWH managed by the Wuchang district Center for Disease Control and Prevention (CDC) were eligible for recruitment. This is because all individuals diagnosed with HIV are reported to the Wuchang CDC through the China National HIV/AIDS Comprehensive Response Information Management System (CRIMS) as required by health protocols in the region.

HIV-negative participants were recruited from the general population in Wuchang. A two-stage cluster random sampling method was employed for this recruitment. First, communities were selected as primary sampling units (PSUs) in the first stage, and families were selected in the second stage. All communities were eligible for certainty PSUs, of which 11 communities were selected with a probability proportional to the sized sampling method. Within each of the 11 communities, 36 households were selected through a systematic random sampling method, and all members of the households received an invitation to participate in the study. To ensure that the age structure of the participants mirrored that of the natural population, we substituted randomly the sample where individuals of an age group were missing.

### Data Collection

All participants provided demographic information which included age and gender. All participants self-reported on COVID-19 testing history, which we double-checked by name and identification card number in the recorded COVID-19 patients' records of the CDC information management system. All SARS-CoV-2 infection diagnoses followed the 8th edition of clinical practice guidelines for COVID-19 in China (8). Information from HIV-negative participants was collected using a structured pretested questionnaire. The PLWH participants provided additional information on chronic co-morbidities, HIV infection route, antiretroviral (ARV) regimen, and current opportunistic infections (OIs) if any. To ensure accuracy, the PLWH data on ARV regimens was re-obtained from the CRIMS.

### Laboratory Procedures

The PLWH received CD4+ T lymphocyte count (CD4 count) and HIV viral load (HIV-VL) tests. All recruited HIV-negative participants received HIV antibody screening tests to ensure that all individuals in the control group were HIV-negative. All participants were tested for the SARS-CoV-2 infection using a throat swab sample SARS-CoV-2 real-time fluorescence polymerase chain reaction (RT-PCR) test and serum IgM/IgG antibody test. All positive tests (RT-PCR, IgM, or IgG positive) were sent to China CDC for confirmation. In the laboratory, the SARS-CoV-2 infection diagnosis was confirmed by respiratory specimens RT-PCR (Shengxiang Biotechnology Co., LTD), serum SARS-CoV-2 IgM/IgG antibody colloidal gold test, and magnetic particle chemiluminescence (qualitative result) (Guangzhou Wanfu Biotech Co., LTD). All test kits used in the study were approved by the China Food and Drug Administration (FDA).

### Definitions

Chronic co-morbidities in this study include hypertension, diabetes, chronic respiratory disease, cancer, and any other clinically diagnosed chronic disease. Our definition of OIs followed the guideline formulated by the United States Department of Health and Human Services (DHHS) (9). All individuals with the SARS-CoV-2 infection were divided into symptomatic patients and asymptomatic infectors. We defined a “symptomatic patient” as a patient diagnosed with clinical manifestations, a positive SARS-CoV-2 nucleic acid test. We divided an asymptomatic infector into nucleic acid-positive asymptomatic infector and serology-positive asymptomatic infector. A “nucleic acid-positive asymptomatic infector” was diagnosed as an infector without clinical manifestations, but had a positive SARS-CoV-2 nucleic acid test. A “serology-positive asymptomatic infector” referred to an infector without clinical manifestations, who had a negative SARS-CoV-2 nucleic acid test, but a positive IgM or IgG antibody examination. Our estimated total SARS-CoV-2 infection rate included the proportions of symptomatic patients, nucleic acid-positive asymptomatic infectors, and serology-positive asymptomatic infectors.

### Statistical Analysis

Continuous variables were expressed as median and interquartile ranges (Q) [M(*P*25, *P*75)], and categorical variables were expressed as frequency and percentages. We compared continuous variables using the Wilcoxon rank-sum test and categorical variables using the χ^2^ test or the Fisher's exact test. The crude rate of the SARS-CoV-2 infection with a 95% confidence interval (95% CI) was estimated using the exact binomial distribution. A logistic regression model was used to assess the difference in the adjusted rate of the SARS-CoV-2 infection among the PLWH and HIV-negative participants. The regression model was adjusted for age, gender, and chronic co-morbidities. Univariate and multivariable modified Poisson regression methods were used to explore the risk factors associated with the SARS-CoV-2 infection among PLWH. A two-sided *p*-value of < 0.05 was deemed statistically significant. STATA version 13.0 (STATA Corporation, College Station, Texas) and IBM SPSS Statistics Version 26.0 (SPSS Corporation, Chicago) software were used for all statistical analysis.

## Results

Overall, 910 PLWH under the management of the Wuchang CDC were eligible for recruitment in the study. A total of two individuals were excluded because they were living abroad during the Wuhan lockdown, and 51 refused to participate in this study. A total of 1,100 HIV-negative individuals selected from the residents living in the Wuchang district were offered participation in the study of which 52 declined. Overall, 857 PLWH and 1,048 HIV-negative individuals were enrolled in this study. The PLWH participants were significantly younger than the HIV-negative participants (*p* = 0.001). Also, the PLWH participants were predominantly males (*p* = 0.001) and had fewer co-morbidities than the HIV-negative participants (*p* = 0.001) (Table 1).

**Table 1:** SARS-CoV-2 infection between HIV positive and negative group in Wuchang District, 2020 (*N* = 1905).

	**HIV-positive group (*N =* 857)**	**HIV-negative group (*N =* 1048)**	***H/χ^2^ value***	***P*-value**
**Age, year** ^*^	39.7 (29, 50)	47.4 (37, 58)	−12.432	0.001
**Gender (%)**			459.156	0.001
Male	774 (90.3)	451 (43.0)		
Female	83 (9.7)	597 (57.0)		
**Chronic Co-morbidities (%)**			118.143	0.001
No	806 (94.1)	793 (75.7)		
Yes	51 (5.9)	255 (24.3)		
**Total SARS-CoV-2 infection (%)**	14 (1.6)	68 (6.5)	26.978	0.001
Symptomatic patients	6 (0.7)	5 (0.5)	0.408	0.523
Asymptomatic infectors				
nucleic acid positive	4 (0.5)	0 (0.0)	4.902	0.040
serology positive	4 (0.5)	63 (6.0)	42.714	0.001
IgM (+) IgG (-)	1 (0.1)	11 (1.1)		
IgM (-) IgG (+)	2 (0.2)	29 (2.7)		
IgM (+) IgG (+)	1(0.1)	23 (2.2)		

* *The data were expressed as median and interquartile ranges (Q) [M (P25, P75)]. Categorical variables were expressed as frequency and percentages Comparisons of continuous variables using the Wilcoxon rank-sum test and categorical variables using the χ^2^ test or the Fisher exact test. “Symptomatic patient” as diagnosed patient with clinical manifestations, a positive SARS-CoV-2 nucleic acid test. “nucleic acid positive asymptomatic infector” were diagnosed infector without clinical manifestations, but had positive SARS-CoV-2 nucleic acid test. “serology positive asymptomatic infector” referred to infector without clinical manifestations, who had a negative SARS-CoV-2 nucleic acid test, but a positive IgM or IgG antibody examination*.

### SARS-CoV-2 Infection Between PLWH and HIV-Negative Group

The crude SARS-CoV-2 infection rate was 1.63% (14/857) among PLWH and 6.49% (68/1048) in the HIV-negative group. Of the 14 SARS-CoV-2-infected PLWH participants, 6 (42.8%) were symptomatic patients, 4 (28.6%) were nucleic acid-positive asymptomatic infectors, and 4 (28.6%) were serology-positive asymptomatic infectors. Among the 68 HIV-negative participants diagnosed with the SARS-CoV-2 infection, 5 (7.4%) were symptomatic patients, and 63 (92.6%) were serology-positive asymptomatic infectors.

The adjusted rate of the SARS-CoV-2 infection was lower among the PLWH participants (1.96, 95% CI: 0.90–3.01) than among the HIV-negative participants (5.74, 95% CI: 4.31–7.17; *p* = 0.001). Similarly, the adjusted rate of the serology-positive asymptomatic infectors was significantly lower among the PLWH participants (0.54, 95% CI: 0.00–1.07) than in the HIV-negative participants (5.46, 95% CI: 4.02–6.91; *p* = 0.001). But the adjusted rate of symptomatic patients did not significantly differ between the PLWH participants (1.10, 95% CI: 0.11–2.10) and HIV-negative participants (0.37, 95% CI: 0.04–0.69; *p* = 0.107) (Table 2). The rate of serology-positive asymptomatic infectors among SARS-CoV-2 infection of PLWH is lower than that in the HIV-negative population (0.4% vs. 6.0%, *p* = 0.001).

**Table 2:** Comparison of SARS-CoV-2 infection between HIV positive and negative group in Wuchang District (*N* = 1905).

	**HIV positive group (*N =* 857)**	**HIV negative group (*N =* 1,048)**	***P*-value**
**Total SARS-CoV-2 infection**			
Crude rate (%, 95% CI)^#^	1.63 (0.78–2.48)	6.49 (4.99–7.98)	
Adjusted rate (%, 95% CI^*^	1.96 (0.90–3.01)	5.74 (4.31–7.17)	0.001
**Symptomatic patients**			
Crude rate (%, 95% CI)^#^	0.70 (0.14–1.26)	0.48 (0.06–0.90)	
Adjusted rate (%, 95% CI)^*^	1.10 (0.11–2.10)	0.37 (0.04–0.69)	0.107
**nucleic acid positive asymptomatic infectors**			
Crude rate (%, 95% CI)^#^	0.47 (0–0.92)	0	
Adjusted rate (%, 95% CI)^*^	NA	NA	NA
**serology positive asymptomatic infectors**			
Crude rate (%, 95% CI)^#^	0.47 (0–0.92)	6.01 (4.57–7.45)	
Adjusted rate (%, 95% CI)^*^	0.54 (0.00–1.07)	5.46 (4.02–6.91)	0.001

# *Confidence intervals estimated using exact binomial distribution*.

* *The adjusted rate was obtained after adjusting for age, gender, and chronic comorbidities using logistic regression*.

### Comparison of the Characteristics of SARS-CoV-2-Infected and Non-Infected PLWH

The PLWH infected with SARS-CoV-2 tended to be much older than uninfected PLWH (53.5 years vs. 35.0 years, *p* = 0.005) and had a higher rate of chronic co-morbidities (*p* = 0.048). In addition, the PLWH with OIs had a higher SARS-CoV-2 infection rate (14.3%) compared to PLWH without OIs (0.6%) (*p* = 0.005). There were no significant differences between the two groups in terms of these factors: ARV regimens, gender, HIV transmission routes, CD4 count, and HIV viral load count (Table 3).

**Table 3:** Demographic features of enrolled PLWH in Wuhan, China, 2020 (*N* = 857).

**Characteristics**	**Uninfected SARS-CoV-2 (*N =* 843)**	**Infected SARS-CoV-2 (*N =* 14)**	***χ^2^*-value**	***P*-value**
**Age, year** ^*^	35.0 (29, 49)	53.5 (42.25, 61)	NA	0.005
**Gender (%)**			0.105	1.000
Male	761 (90.3)	13 (92.9)		
Female	82 (9.7)	1 (7.1)		
**Chronic comorbidities (%)**			5.892	0.048
No	794 (94.2)	11 (78.6)		
Yes	49 (5.8)	3 (21.4)		
**The transmission route of HIV (%)**			0.202	0.904
Heterosexual transmission	196 (23.3)	3 (21.4)		
Homosexual	637 (75.5)	11 (78.6)		
Other	10 (1.2)	0 (0.0)		
**OIs (%)**			31.871	0.005
Yes	5 (0.6)	2 (14.3)		
No	838 (99.4)	12 (85.7)		
**CD4 count (%)**			5.467	0.074
<100 cells/μL	26 (3.1)	2 (14.3)		
≥100 cells/μL	817 (96.9)	12 (85.7)		
**HIV-VL (%)**			0.025	1.000
<20 cells/μL	618 (73.3)	10 (71.4)		
≥20 cells/μL	225 (26.7)	4 (28.6)		
**ARV regimens (%)**			4.428	0.219
NRTI+NNRTI	699 (82.9)	13 (92.9)		
PIs-based	78 (9.3)	0 (0.0)		
INIs-based	51 (6.0)	0 (0.0)		
Not on ARV	15 (1.8)	1 (7.1)		

* *Data were expressed as median and interquartile ranges (Q) [M (P25, P75)]. Categorical variables were expressed as frequency and percentage. Comparisons of continuous variables were assessed using the Wilcoxon rank-sum test, while categorical variables were assessed using the χ2 test or the Fisher exact test. NRTI, nucleoside reverse transcriptase inhibitors. NNRTI, non-nucleoside reverse transcriptase inhibitors. PIs, protease inhibitors. INIs, integrase inhibitors. OIs, opportunistic infections*.

### Risk Factors of SARS-CoV-2 Infection Among PLWH

The univariate regression analysis results showed that older age ≥50 years (OR = 8.36, 95% CI: 3.01–23.22, *p* = 0.001), chronic co-morbidities (OR = 4.70, 95% CI: 1.42–15.58, *p* = 0.011), opportunistic infections (OR = 23.05, 95% CI: 5.93-89.57, *p* = 0.001), and CD4 count <100/μl (OR = 0.19, 95% CI: 0.05–0.76, *p* = 0.019) were associated with increased odds of SARS-CoV-2 infection among PLWH. In the multivariable regression analysis, only older age ≥50 years (aOR = 4.50, 95% CI: 1.34–15.13, *p* = 0.015) and opportunistic infections (aOR = 9.59, 95% CI: 1.54–59.92, *p* = 0.016) were associated with increased risks of SARS-CoV-2 infection among PLWH. The model was adjusted for gender, chronic co-morbidities, the transmission route of HIV, CD4 count, HIV viral load count, and ARV regimens (Table 4).

**Table 4:** The risk factors of SARS-CoV-2 infection among PLWH from Wuchang district in Wuhan, China (*N* = 857).

**Characteristics**	**Univariate analysis**		Multivariable analysis^*^	
	**OR (95%CI)**	***P*-value**	**Adjusted OR (aOR) (95%CI)**	***P*-value**
**Age (years)**				
18–49	1.00		1.00	
≥50	8.36 (3.01–23.22)	0.001	4.50 (1.34–15.13)	0.015
**Gender**				
Male	1.00		1.00	
Female	0.65 (0.12–3.50)	0.616	0.82 (0.07–9.12)	0.872
**Chronic Co-morbidities**				
No	1.00		1.00	
Yes	4.70 (1.42–15.58)	0.011	2.17 (0.52–9.12)	0.290
**The transmission route of HIV**				
Non-MSM	1.00		1.00	
MSM	0.36 (0.11–1.19)	0.093	0.53 (0.13–2.11)	0.617
**OIs**				
No	1.00		1.00	
Yes	23.05 (5.93-89.57)	0.001	9.59 (1.54-59.92)	0.016
**CD4 count (cells/μL)**				
<100	1.00		1.00	
≥100	0.19 (0.05–0.76)	0.019	0.27 (0.04–1.96)	0.197
**HIV-VL (copies/mL)**				
<20	1.00		1.00	
≥20	1.38 (0.46–4.17)	0.567	0.98 (0.26–3.79)	0.985
**ARV regimens**				
Yes	1.00		1.00	
No	0.66 (0.04–11.00)	0.769	1.00 (1.00–1.00)	NA

*MSM, men who have sex with men*.

* *Each association was mutually adjusted for the other characteristics in the table. OIs, opportunistic infections*.

## Discussion

This study extends the existing literature by our consideration of all three types of SARS-CoV-2 infection and investigated the risks of total SARS-CoV-2 infection among PLWH. A cross-sectional survey conducted in May 2020 (1 month after the primary onset of the SARS-CoV-2 epidemic was contained in China), showed that the rate of positive SARS-CoV-2 antibodies among the Wuhan populations was 4.43% (10). This finding is similar to the total rate of SARS-CoV-2 infection observed among the HIV-negative participants in our study (6.5%). Our results also showed that the rate of SARS-CoV-2 infection was lower among PLWH (1.6%) than among the HIV-negative participants. But the infected PLWH participants exhibited more varied forms of SARS-CoV-2 infections than the infected HIV-negative participants. The 0.45% rate of symptomatic SARS-CoV-2 infections reported in Wuhan (3) was similar to the rates observed among both infected HIV-negative participants (0.5%, 5/1,048) and infected PLWH (0.7%, 6/857) in our study. Similarly, findings from another study showed no significant difference in the rate of symptomatic patients between the PLWH and HIV-negative populations (11).

We found that more SARS-CoV-2-infected PLWH tended to be nucleic acid-positive asymptomatic infectors than SARS-CoV-2 infected HIV-negative individuals. Although no nucleic acid-positive asymptomatic infectors were found in HIV-negative participants in this study, we observed that 0.5% (4/857) rate of nucleic acid-positive asymptomatic infectors among SARS-CoV-2-infected PLWH was higher than the previous rates of 0.013% (8/61,437 in Wuchang district) and 0.001% (221/1,58,403 in Wuhan city) reported by previous Wuhan studies (12, 13). We hypothesize that two potential factors may have contributed to this finding. First, it is possible that immune deficiency causes the body of PLWH to clear the SARS-CoV-2 antibodies more slowly than SARS-CoV-2-infected HIV-negative individuals (14–16). This explanation is possible as a previous study found a median virus shedding time of 19 days in asymptomatic infectors and 14 days in symptomatic patients among SARS-CoV-2 infected HIV-negative individuals (14). On the other hand, another study found an-18 day median time of virus shedding among 68% of the SARS-CoV-2 infected PLWH, but also observed that the virus was still detectable in 32% of the patients 40 days later (17). Second, PLWH may not exhibit typical clinical symptoms of immunodeficiency with a SARS-CoV-2 infection due to their compromised immunity (18, 19). This may have increased their possibility of being nucleic acid-positive asymptomatic infectors at the initial stages of a SARS-CoV-2 infection.

We also found that serology-positive asymptomatic infectors were preponderant among SARS-CoV-2-infected HIV-negative individuals (6.0%, 63/1,048) than SARS-CoV-2-infected PLWH (0.5%, 4/857). This outcome was salient even when multivariable regression models were adjusted for possible confounding factors including age and gender. We speculate that three factors concurrently or individually may have potentially accounted for this finding. First, previous study findings have suggested that B-cell dysfunction appears during an HIV infection and results in impaired antibody responses to vaccines (20). Thus, the compromised immunity of PLWH leads to insufficient antibody production than found in HIV-negative people. Second, it is possible that serum levels of IgG antibody decrease faster in PLWH than in HIV-negative populations. A study in the Chongqing province of China made a similar observation that antibodies decreased by more than 70% in 90% of the SARS-CoV-2-infected HIV-negative populations after 2 months (14). Our previous findings also showed that the positive IgG conversion rate for SARS-CoV-2 infection was relatively lower and quickly lost in PLWH (21). Finally, practicing preventive health behaviors like social distancing could have shielded PLWH from SARS-CoV-2 infection.

Our results showed that PLWH with OIs is at higher risk of SARS-CoV-2 infection than PLWH without OIs. It is a well-known phenomenon that the appearance of OIs means severely impaired immunity, and that means the affected PLWH are prone to getting other infections (22). However, reports about SARS-CoV-2 infection in PLWH with OIs are limited (23). On the other hand, the common OIs among PLWH include tuberculosis, pneumocystis pneumonia, and bacterial pneumonia. All of these diseases could compromise the immunity of the local pulmonary and cause lung damage in severe cases (24). Possibly, the compromised immunity of the local pulmonary could enhance the risk of SARS-CoV-2 infection in theory. Some studies have also suggested that PLWH with tuberculosis infection is more susceptible to SARS-CoV-2 infection (25, 26). However, more studies are needed to ascertain the role of these factors in SARS-CoV-2 infection among PLWH.

At the early onset of the SARS-CoV-2 pandemic outbreak, many scholars speculated that the ARV drugs may have therapeutic and preventive effects on SARS-CoV-2 infection (27, 28). Yet, a study in Spain found that the ARV drugs could not reduce SARS-CoV-2 infection-related morbidity among PLWH (29). Findings from a randomized controlled open-label trial also showed no benefits in the use of lopinavir-ritonavir among the SARS-CoV-2 infection patients (30). Our study findings also suggest that the ARV drugs do not provide prophylaxis for SARS-CoV-2 infection among PLWH. Thus, the speculated protection that ARVs offer to PLWH against SARS-CoV-2 infection is unfounded; hence we recommend that routine SARS-CoV-2 testing interventions should be tailored to include PLWH.

Our study has several limitations. First, this was a cross-sectional study, and hence, may not reflect the conditions at the early onset of the SARS-CoV-2 pandemic outbreak in Wuhan. Second, our study sample size is relatively small, and this limited us from conducting more significant analyses. In addition, differences in the adjusted rate of asymptomatic SARS-CoV-2 infections between the two groups could not be compared since we found no asymptomatic patients among our HIV-negative participants. Finally, although the serological IgM/IgG antibody test had some false positives, each positive specimen was double-tested to reduce the risk of this error.

In conclusion, our study findings show that SARS-CoV-2 infected PLWH are more likely to be nucleic acid-positive asymptomatic infectors, and the seroprevalence of antibodies is lower among SARS-CoV-2-infected PLWH than SARS-CoV-2-infected HIV-negative individuals. Therefore, strategies should be established to enable routine SARS-CoV-2 testing among PLWH and facilitate early diagnosis among the population. We also found that older PLWH and those with OIs are at higher risk of SARS-CoV-2 infection. Therefore, more attention should be given to encouraging the practice of personal protective behaviors (like hand washing and social distancing) by this group of PLWH to reduce exposure to infection.

## Data Availability Statement

The original contributions presented in the study are included in the article/supplementary material, further inquiries can be directed to the corresponding author/s.

## Ethics Statement

The studies involving human participants were reviewed and approved by Medical Ethics Committee Zhongnan Hospital of Wuhan University. The patients/participants provided their written informed consent to participate in this study.

## Author Contributions

WT and KL conceived and designed this investigation. FM and XZ helped to design the scheme of the investigation. FM and MW collected the original data. MW and SW analyzed the data. MW, WT, and KL contributed to the interpretation of the data. MW, SW, GM, WT, and KL contributed to the writing of the paper. All authors have read and approved the final manuscript.

## Funding

This work was supported by the National Key Research and Development Program of China (2017YFE0103800), the National Nature Science Foundation of China (81903371), NIMH (R34MH119963), the National Science and Technology Major Project (2018ZX10101-001-001-003), Special Found on Prevention and Control of New Coronary Pneumonia in Guangdong Universities (2020KZDZX1047), Medical Science and Technology Innovation Platform Support Project of Zhongnan Hospital, Wuhan University (PTXM2020008), Science and Technology Innovation Cultivation Fund of Zhongnan Hospital, Wuhan University (cxpy2017043), and Medical Science Advancement Program (Basic Medical Sciences) of Wuhan University (TFJC2018004).

## Conflict of Interest

The authors declare that the research was conducted in the absence of any commercial or financial relationships that could be construed as a potential conflict of interest.

## Publisher's Note

All claims expressed in this article are solely those of the authors and do not necessarily represent those of their affiliated organizations, or those of the publisher, the editors and the reviewers. Any product that may be evaluated in this article, or claim that may be made by its manufacturer, is not guaranteed or endorsed by the publisher.
