# Corrigendum: Risk of SARS-CoV-2 infection among people living with HIV in Wuhan, China

**DOI:** 10.3389/fpubh.2024.1494917

**Published:** 2024-09-24

**Authors:** Mengmeng Wu, Fangzhao Ming, Songjie Wu, Yanbin Liu, Xiaoxia Zhang, Wei Guo, Gifty Marley, Weiming Tang, Ke Liang

**Affiliations:** ^1^Department of Infectious Diseases, Zhongnan Hospital of Wuhan University, Wuhan, China; ^2^Wuhan Research Center for Infectious Diseases and Cancer, Chinese Academy of Medical Sciences, Wuhan, China; ^3^Wuchang District Center for Disease Control and Prevention, Wuhan, China; ^4^Department of Nosocomial Infection Management, Zhongnan Hospital of Wuhan University, Wuhan, China; ^5^Department of Pathology, Zhongnan Hospital of Wuhan University, Wuhan, China; ^6^Department of Pathology, School of Basic Medical Sciences, Wuhan University, Wuhan, China; ^7^School of Public Health of Nanjing Medical University, Nanjing, China; ^8^Guangdong Second Provincial General Hospital, Guangzhou, China; ^9^University of North Carolina at Chapel Hill Project-China, Guangzhou, China; ^10^Hubei Engineering Center for Infectious Disease Prevention, Control and Treatment, Wuhan, China

**Keywords:** SARS-CoV-2, people living with human immunodeficiency virus (PLWH), IgG, IgM, asymptomatic infectors, symptomatic patient

In the published article “Risk of SARS-CoV-2 Infection Among People Living With HIV in Wuhan, China,” there was an error in affiliation #8, Instead of “Guangdong Second Provincial Central Hospital, Southern Medical University, Guangzhou, China,” it should be “Guangdong Second Provincial General Hospital, Guangzhou, China.”

The authors apologize for this error and state that this does not change the scientific conclusions of the article in any way. The original article has been updated.

